# Effects of Ergot Alkaloids on Liver Function of Piglets as Evaluated by the ^13^C-Methacetin and ^13^C-α-Ketoisocaproic Acid Breath Test

**DOI:** 10.3390/toxins5010139

**Published:** 2013-01-15

**Authors:** Sven Dänicke, Sonja Diers

**Affiliations:** Institute of Animal Nutrition, Friedrich-Loeffler-Institute (FLI), Federal Research Institute for Animal Health, Braunschweig D-38116, Germany

**Keywords:** ergot alkaloids, breath test, pig

## Abstract

Ergot alkaloids (the sum of individual ergot alkaloids are termed as total alkaloids, TA) are produced by the fungus *Claviceps purpurea*, which infests cereal grains commonly used as feedstuffs. Ergot alkaloids potentially modulate microsomal and mitochondrial hepatic enzymes. Thus, the aim of the present experiment was to assess their effects on microsomal and mitochondrial liver function using the ^13^C-Methacetin (MC) and ^13^C-α-ketoisocaproic acid (KICA) breath test, respectively. Two ergot batches were mixed into piglet diets, resulting in 11 and 22 mg (Ergot 5-low and Ergot 5-high), 9 and 14 mg TA/kg (Ergot 15-low and Ergot 15-high) and compared to an ergot-free control group. Feed intake and live weight gain decreased significantly with the TA content (*p* < 0.001). Feeding the Ergot 5-high diet tended to decrease the 60-min-cumulative ^13^CO_2_ percentage of the dose recovery (cPDR_60_) by 26% and 28% in the MC and KICA breath test, respectively, compared to the control group (*p* = 0.065). Therefore, both microsomal and mitochondrial liver function was slightly affected by ergot alkaloids.

## 1. Introduction

Members of the fungal family of *Clavicipitaceae* infest grass species, including cereal grains, and are capable of producing a number of so-called ergot alkaloids, which might exert adverse effects on livestock [1]. For monogastric animals, such as pigs, especially the species *Claviceps purpurea *is of special interest, as it infects mainly rye, wheat and triticale and develops mycelium instead of kernels on the ears. These mycelia appear as discolored hardened sclerotia and might contain a total alkaloid (TA) content between 0.01% and 0.21% composed of individual alkaloids. These alkaloids potentially interact with adrenergic, serotoninergic and dopaminergic receptors, depending on their chemical nature, concentration and proportion to each other [1]. Besides their pharmacological potential, ergot alkaloids are also capable of influencing the liver, the organ exposed to these substances via the portal vein directly after absorption from the gastro-intestinal tract. The liver is involved in xenobiotic metabolism and was shown to respond to ergocryptine and ergometrine maleate with an increased liver weight and an altered glycogen metabolism of rats [2,3,4]. Moreover, the xenobiotic metabolizing enzymes of the cytochrome P450 (CYP) family were demonstrated to be involved in ergot metabolism [5] and to be induced [6]. Moreover, hepatic mitochondrial Ca^2+^ ATPase activity and, consequently, ATP formation was found to be inhibited by ergotamine and ergonovine [7]. It becomes clear that the hepatocyte might be affected, at least at the cytosolic, microsomal and mitochondrial level. Each of these hepatic compartment functions can be tested by non-invasive breath tests using specific carbon-labeled compounds (for reviews, see [8,9,10,11,12,13,14]). These compartment-specific substrates are primarily metabolized to CO_2 _and include, for example, ^13^C-α-ketoisocaproic acid (KICA), *N*-[4-Methoxyphenyl]acetamid (^13^C-Methacetin, MC) and ^13^C-phenylalanine for testing mitochondrial, microsomal and cytosolic compartments, respectively (e.g., [12]). Effects of ergot on pigs are rather inconsistent [15] and might be due to the mentioned variation in ergot alkaloid content and pattern [16]. So far, effects on porcine livers were only evaluated by blood clinical-chemical measures, indicative of hepatocyte function and integrity [17,18]. However, these parameters are static and cannot be regarded as reliable markers for global liver function or as a tool to quantify functional hepatic reserve [12]. Thus, the aim of the present experiment was to test the effects of two ergot batches differing in alkaloid content and pattern on feed intake, weight gain and serum clinical-chemical measures of rearing piglets, along with measurement of the hepatic mitochondrial and microsomal function using the KICA and MC breath test, respectively.

## 2. Results

### 2.1. Chemical Composition of Ergot and Diets

The ergot batches did not differ markedly in crude nutrients and contained on average approximately 20% crude protein, 31% crude fat and 23% crude fiber (Table 1). In contrast, ergot batches differed markedly in total alkaloid content. While Batch 5 contained 1381 mg total alkaloids/kg ergot, the corresponding concentration of Ergot Batch 15 amounted to 436 mg/kg ergot (Table 1). The proportions of key alkaloids of total alkaloids were 58% and 62% for Ergot Batches 5 and 15, respectively (Table 1). Related to the respective total alkaloids, the most abundant individual alkaloids were ergotamine (26%) and ergocristine (28%) in Ergot Batches 15 and 5, respectively. Both batches contained similar proportions of ergocornine and ergocorninine. Ergot Batch 5 contained more ergometrine, ergometrinine, ergocristinine, ergosine and ergosinine, but less ergotamine, ergotaminine, ergocryptine and ergocryptinine than Ergot Batch 15. 

**Table 1:** Chemical composition of the ergot batches.

	Ergot 5 ^1^	Ergot 15 ^2^
Crude nutrients [g/kg] ^3^		
Crude ash	27.7	28.8
Crude protein	213.5	183.7
Crude fat	300.3	321.8
Crude fiber	253.1	203.8
Starch	35.7	32.9
Sugar	5.3	14.2
Fatty acid composition [g/100 g crude fat]		
Caprylic acid (C8:0)	0.0	1.4
Lauric acid (C12:0)	1.8	2.8
Myristic acid (C14:0)	0.6	0.5
Palmitic acid (C16:0)	32.6	30.2
Palmitoleic acid (C16:1)	2.7	3.3
Stearic acid (C18:0)	9.0	7.8
Oleic acid (C18:1)	19.7	20.6
Linoleic acid (C18:2)	14.8	18.2
Linolenic acid (C18:3)	0.2	0.4
Arachidic acid (C20:0)	1.3	1.2
Behenic acid (C22:0)	0.4	0.3
Erucic acid (C22:1)	0.1	0.2
Ricinoleic acid (12-OH-C18:1)	15.2	7.4
Lignoceric acid (C24:0)	0.2	0.1
Alkaloids [mg/kg]^ 3^		
Total alkaloids ^4^	1381.1	435.8
Key alkaloids ^5^	794.8	271.4
Ergometrine	149.0	31.9
Ergometrinine	62.1	7.5
Ergotamine	125.0	113.4
Ergotaminine	23.6	38.0
Ergocornine	79.3	23.7
Ergocorninine	35.0	10.2
Ergocristine	381.9	80.1
Ergocristinine	91.5	22.3
Ergocryptine	59.5	22.3
Ergocryptinine	18.9	19.9
Ergosine	303.9	54.8
Ergosinine	51.3	11.7

^1 ^Ergot batch contained 100% ergot; ^2 ^Ergot batch contained 45.2% ergot and 54.8% rye; ^3 ^Based on a dry matter content of 880 g/kg; ^4 ^Sum of ergometrine, ergotamine, ergocornine, ergocristine, ergocryptine, ergosine and of their -inine isomers; ^5 ^Sum of ergometrine, ergotamine, ergocornine, ergocristine, ergocryptine.

Fatty acid profiles of both ergot batches were comparable, except ricinoleic acid (12-OH–C18:1). Its proportion of total fatty acid methyl esters amounted to 15% and 7% for Ergot Batches 5 and 15, respectively. Further abundant fatty acids were palmitic acid (C16:0), palmitoleic acid (C16:1), stearic acid (C18:0), oleic acid (C18:1) and linoleic acid (C18:2), with average proportions of 31%, 3%, 8%, 20% and 17%, respectively. 

The measured total alkaloid concentrations deviated from the targeted 8.4 mg/kg by +26% and +8% for Ergot 5-low and Ergot 15-low, respectively, whilst the deviations from the higher target alkaloid concentration of 16.8 mg/kg amounted to +32% and −20% for Ergot 5-high and Ergot 15-high diets, respectively (Table 2).

**Table 2:** Composition of experimental diets (g/kg).

	Control	Ergot 5-low	Ergot 5-high	Ergot 15-low	Ergot 15-high
Components					
Barley	215.2	209.2	203.2	197	178.8
Wheat	200	200	200	200	200
Maize	150	150	150	150	150
Soybean meal	200	200	200	200	200
Soybean concentrate ^1^	40	40	40	40	40
Hydrothermally treated maize	100	100	100	100	100
Soybean oil	40	40	40	40	40
Vitamin and mineral premix ^2^	40	40	40	40	40
DL-methionine	2.5	2.5	2.5	2.5	2.5
L-lysine mono-hydro-chloride	4.5	4.5	4.5	4.5	4.5
L-threonine	2.0	2.0	2.0	2.0	2.0
L-tryptophan	0.5	0.5	0.5	0.5	0.5
Formic acid ^3^	5.0	5.0	5.0	5.0	5.0
Phytase ^4^	0.3	0.3	0.3	0.3	0.3
Ergot Batch 5 ^5^		6.0	12.0		
Ergot Batch 15 ^6^				18.2	36.4
Calculated composition ^7^					
Total alkaloids ^8^ [mg/kg]		8.4	16.8	8.4	16.8
Crude protein ^9^	184	184	185	185	187
Metabolizable energy (ME)[MJ/kg] ^9^	14.0	14.0	14.0	14.0	14.0
Lysine ^9^	12.3	12.3	12.4	12.4	12.6
Methionine and cystine ^9^	7.4	7.4	7.4	7.5	7.5
Threonine ^9^	8.1	8.1	8.1	8.2	8.2
Tryptophan ^9^	2.6	2.6	2.6	2.7	2.7
Calcium ^9^	9.3	9.3	9.4	9.5	9.6
Total phosphorus ^9^	5.9	5.9	5.9	5.9	6.0
Sodium ^9^	2.2	2.3	2.3	2.3	2.3
Analyzed composition^ 7^					
Crude ash	64.0	60.3	61.6	58.3	62.9
Crude protein	212	197	199	191	200
Crude fat	48.6	40.7	51.1	54.8	41.6
Crude fiber	34.9	33.1	34.4	37.0	36.6
ME ^2^ [MJ/kg]	13.6	13.4	13.8	13.9	13.6
Total alkaloids ^8^ [mg/kg]	<d.l.	10.6	22.1	9.1	13.5
Key alkaloids ^10^ [mg/kg]	<d.l.	5.3	11.1	5.1	7.5
Ergocristine [μg/kg]	<d.l.	1848.0	4278.6	1592.6	2100.6
Ergocornine [μg/kg]	<d.l.	525.0	1098.5	348.0	584.2
Ergocryptine [μg/kg]	<d.l.	281.1	626.7	445.2	663.2
Ergotamine [μg/kg]	<d.l.	900.2	1900.1	1913.9	3025.8
Ergometrine [μg/kg]	<d.l.	1714.2	3185.3	818.1	1135.5
Ergosine [μg/kg]	<d.l.	1910.6	4240.0	889.5	1566.1
∑inine-isomeres [μg/kg] ^11^	<d.l.	3376.3	6840.9	3057.6	4404.7

^1^ Soycomil^®^, 650 g crude protein/kg, Denkavit Ingredients, Warendorf, Germany;^ 2^ Provided per 1 kg premix: 240 g Ca; 60 g P; 55 g Na; 10 g Mg; 400,000 I.E. vitamin A; 40,000 I.E. vitamin D_3_; 1200 mg vitamin E; 37.5 mg vitamin B_1_; 100 mg vitamin B_2_; 100 mg vitamin B_6_; 750 mg vitamin B_12_; 52.5 mg vitamin K_3_; 500 mg nicotinic acid; 337.5 mg Ca-panthotenate; 5000 mg cholin chloride; 4000 mg Fe; 1000 mg Cu; 2000 mg Mn; 4000 mg Zn; 50 mg J; 15 mg Se; 20 mg Co; ^3^ACIDOMIX^®^ Formic 65 G, Röthel GmbH Schwänheit 10, D-34281 Gudensberg, Deutschland;^ 4 ^ZY PHYTASE 5000 (LOHMANN ANIMAL HEALTH GmbH & Co. KG Heinz-Lohmann-Straße 4 27472 Cuxhaven Deutschland) declared phytase activity (EC 3.1.3.26): 5000 FYT/g;^ 5 ^Ergot batch contained 100% ergot; ^6 ^Ergot batch contained 45.2% ergot and 54.8% rye; ^7^ Based on a dry matter content of 880 g/kg;^ 8 ^Sum of ergometrine, ergotamine, ergocornine, ergocristine, ergocryptine, ergosine and of their –inine isomers; ^9^ Based on table values (DLG-Futterwerttabelle, 1991);^ 10 ^Sum of ergometrine, ergotamine, ergocornine, ergocristine, ergocryptine;^ 11^ Sum of the -inine isomers ergometrinine, ergocorninine, ergotaminine, α-ergocryptinine, ergosinine and ergocristinine. d.l.: detection limit.

### 2.2. Performance

Feed intake was significantly depressed by 23% to 34% by feeding the ergot-containing diets compared to the control group irrespective of ergot batch and dosage (Table 3). The decrease in live weight gain corresponded to the feed intake depression, although the extent of the adverse ergot effect was even more pronounced and reached a proportional decrease between 28% and 76%. 

Based on the feed intake and the live weight of the piglets averaged per group over the whole experimental period of five weeks and the analyzed TA contents of the experimental diets, the mean daily TA exposure of the control, Ergot 5-low, Ergot 5-high, Ergot 15-low and Ergot 15-high group were calculated to be 0.0 (TA content < d.l.), 0.248, 0.586, 0.243 and 0.364 mg/kg body weight, respectively. 

As ergot-related decreases in feed intake and live weight gain were different, the resulting feed-to-gain ratio was not fully compensated and, hence, also increased (Table 3). However, significance was only reached for piglets fed the Ergot 5-high diet when the first week of experiment and the whole experiment were considered.

**Table 3:** Performance of weaned piglets fed a control diet without ergot or diets adjusted to a total ergot alkaloid content of 8.4 mg/kg diet (Ergot 5-low; Ergot 15-low) or 16.8 mg/kg diet (Ergot 5-high; Ergot 15-high) (*n* = 4 pens per treatment with two male and two female piglets in each pen).

Diet	Feed intake (g/d)			Live weight gain (g/d)			Feed to gain ratio (g/g)		
	day 1–7	day 1–21	day 1–35	day 1–7	day 1–21	day 1–35	day 1–7	day 1–21	day 1–35
Control	276 ^a^	425 ^a^	586 ^a^	231 ^a^	335 ^a^	437 ^a^	1.20 ^a^	1.28	1.33 ^a^
Ergot 5-low	230 ^ab^	315 ^b^	446 ^b^	118 ^b^	203 ^b^	313 ^b^	2.06 ^ab^	1.60	1.44 ^ab^
Ergot 5-high	204 ^b^	289 ^b^	385 ^b^	56 ^b^	166 ^b^	238 ^b^	4.51 ^b^	1.89	1.63 ^b^
Ergot 15-low	193 ^b^	306 ^b^	424 ^b^	86 ^b^	200 ^b^	286 ^b^	2.50 ^ab^	1.54	1.49 ^ab^
Ergot 15-high	209 ^b^	321 ^b^	449 ^b^	96 ^b^	212 ^b^	304^b^	2.30 ^ab^	1.51	1.48 ^ab^
ANOVA (*p*-values)									
Diet	0.002	<0.001	<0.001	<0.001	<0.001	<0.001	0.034	0.149	0.023
Sex ^1^	-	-	-	0.397	0.683	0.579	-	-	-
Diet × sex	-	-	-	0.271	0.438	0.735	-	-	-
PSEM	12	15	17	28	36	41	0.65	0.15	0.05

Abbreviations: PSEM = pooled standard error of means; ^1 ^Sex: Female/male; ^ab^ Values with no common superscripts are significantly different within columns (*p* < 0.05).

### 2.3. Clinical-Chemical Characteristics

None of the measured enzyme activities were influenced by dietary treatments (Table 4), and average values were lower than the reference values for piglets of 35 U aspartate aminotransferase (ASAT) per L, 68 U alanine-aminotransferase (ALAT) per L, 4 U glutamate dehydrogenase (GLDH) per L and 45 U Gamma-glutamyltransferase (GGT) per L [19]. Similarly, the serum protein concentration remained uninfluenced by dietary treatments, and the upper reference protein concentration of 86 g/L was not exceeded. The albumin concentration was characterized by a significant interaction between diet and sex and was caused by the significantly higher albumin concentrations of male piglets fed the control and Ergot 5-high diets, while the opposite was observed after feeding the Ergot 15-high diet. Although significant dietary effects were observed, the corresponding reference value of 31 g/L was not exceeded. Total bilirubin concentration was significantly higher in male piglets of the control group compared to all other groups, including the female piglets of the same feeding group. The latter still showed a significantly higher total bilirubin concentration than all piglets of the Ergot 5-high group and male piglets of the Ergot 15-high group. The significant diet and interaction effects for the serum glucose concentration were related to the significantly higher values measured for the male and female piglets fed the Ergot 5-high and 15-high diet compared to the opposite sex of the same feeding groups. Glucose concentrations in serum of the control group did not show such marked sex effects. Glucose concentrations were partly lower than the reference range of 3.9–6.4 mmol/L.

**Table 4:** Clinical-chemical characteristics of weaned piglets fed a control diet without ergot (Control) or diets adjusted to a total ergot alkaloid content of 16.8 mg/kg (Ergot 5-high; Ergot 15-high) (week 4 of experiment, *n* = 8).

Diet	Sex	Protein (g/L)	Albumin (g/L)	Total bilirubin (μmol/L)	Glucose (mmol/L)	ASAT (U/L)	ALAT (U/L)	GLDH (U/L)	GGT (U/L)
Control	Male	47.5	27.0 ^ab^	4.6 ^c^	3.0 ^ab^	24.0	21.5	2.3	17.2
Control	Female	48.2	25.2 ^ab^	3.7 ^b^	3.1 ^abc^	27.3	20.9	2.5	19.3
Ergot 5-high	Male	48.0	27.8 ^b^	1.3^a^	4.2 ^d^	23.1	24.6	1.6	21.3
Ergot 5-high	Female	47.0	23.5 ^a^	1.3 ^a^	3.7 ^cd^	25.2	25.8	1.9	17.9
Ergot 15-high	Male	46.2	25.3 ^ab^	2.5 ^ab^	2.8 ^a^	24.9	24.9	2.0	17.6
Ergot 15-high	Female	45.7	27.6 ^b^	2.0 ^a^	3.6 ^bcd^	17.9	23.4	1.5	19.3
ANOVA (*p*-values)									
Diet		0.475	0.828	0.001	0.002	0.362	0.106	0.276	0.777
Sex		0.848	0.226	0.650	0.539	0.824	0.834	0.893	0.935
Diet × sex		0.877	0.031	0.834	0.034	0.176	0.737	0.620	0.298
PSEM		1.5	1.2	0.5	0.2	2.8	1.7	0.4	1.8

Abbreviations: GGT = Gamma-glutamyl transferase; ASAT = aspartat-aminotransferase; ALAT = alanine-aminotransferase; GLDH = glutamat-dehydrogenase; PSEM = pooled standard error of means; MC = ^13^C-methacetin; KICA = ^13^C-ketoisocaproate; ^ab^ Values with no common superscripts are significantly different within columns (*p* < 0.05).

### 2.4. Breath Test

**Figure 1:**
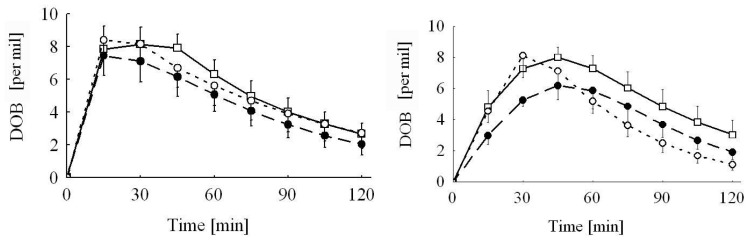
^13^CO_2 _excretion curves of piglets fed the Control diet (―□―) or diets adjusted to a total ergot alkaloid content of 16.8 mg/kg diet (– –●– –, Ergot 5-high; ---○---, Ergot 15-high) (week 3 of experiment, *n* = 8). Piglets were dosed either with 2 mg ^13^C-methacetin (left) or with 2 mg ^13^C-ketoisocaproate (right) per kg live weight orally. ^13^CO_2 _excretion is expressed as delta over baseline (DOB).

**Figure 2:**
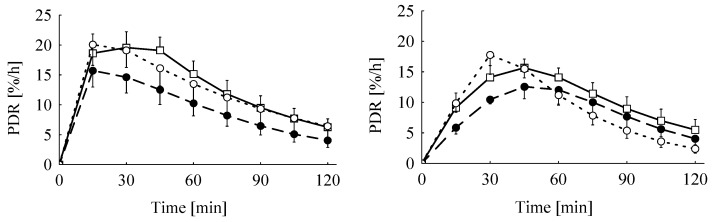
Mean fractional percentage of the applied ^13^C-methacetin (left) and ^13^C-ketoisocaproate (right) dose recovered (PDR) of piglets fed the control diet (―□―) or diets adjusted to a total ergot alkaloid content of 16.8 mg/kg diet (– –●– –, Ergot 5-high; ---○---, Ergot 15-high) (week 3 of experiment, *n *= 8).

**Figure 3:**
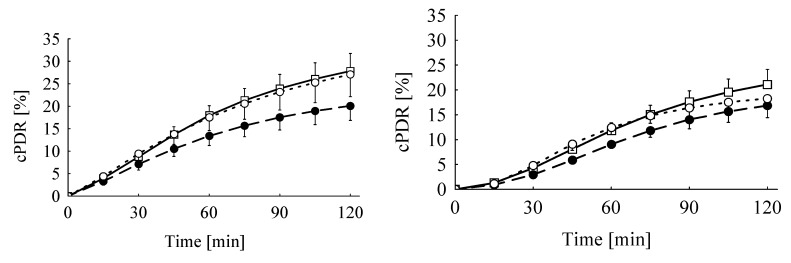
Mean cumulative percentage of the applied ^13^C-methacetin (left) and ^13^C-ketoisocaproate (right) dose recovered (cPDR) of piglets fed the Control diet (―□―) or diets adjusted to a total ergot alkaloid content of 16.8 mg/kg diet (– –●– –, Ergot 5-high; ---○---, Ergot 15-high) (week 3 of experiment, *n* = 8).

Live weight and live weight gain at the day of breath test were only slightly influenced by dietary treatments (*p* = 0.056 and *p* = 0.069, respectively). Compared to the control group, the decrease amounted to 16% and 12% for the Ergot 5-high and 15-high group, respectively, irrespective of sex for live weight, and to 33% and 20% for live weight gain, respectively (Table 5). None of the parameters of the breath test was influenced by feeding the ergot-containing diets or by sex. Exhalation kinetics of ^13^CO_2_ were significantly influenced by the test substrate. The time at the maximum ^13^C-exhalation (*t*_max_) occurred approximately 18 min earlier when MC was used as a test substrate compared to KICA (21 min *vs.* 39 min), while the corresponding maximum delta over base line (DOB) values (DOB_max_) were not significantly different (9.6% *vs.* 8.3 ‰) (Figure 1 and Figure 2, Table 5). Furthermore, the time when the half of the total recovered ^13^CO_2_ was exhaled (*t*_0.5_) was influenced by substrate in a sex-specific manner, as *t*_0.5_ was estimated at 125 min in male piglets given KICA, while all other piglets, independent of sex and applied substrate, had comparable *t*_0.5_, varying from 49 to 86 min (Table 5). The cumulative ^13^CO_2_-recovery (cPDR = cumulative percentage dose recovery) was significantly lower 30 and 60 min after giving the KICA bolus compared to the MC bolus, while after 120 min, differences failed to reach significance (Table 5, Figure 3). Relatively, for piglets given the KICA bolus. The cPDR_30_, cPDR_60 _and the cPDR_120_ were 51%, 29% and 19% lower, respectively, than the corresponding values obtained after MC administration.

**Table 5:** Results of the ^13^C-methacetin (MC) and ^13^C-ketoisocaproate (KICA) breath test of weaned piglets fed a control diet without ergot (Control) or diets adjusted to a total ergot alkaloid content of 16.8 mg/kg diet (Ergot 5-high; Ergot 15-high) (week 3 of experiment, *n* = 4).

Diet	Sex	Substrate	*t*_max_ (min)	DOB_max_ (‰)	*t*_0.5 _(min)	cPDR_30_ (%)	cPDR_60_ (%)	cPDR_120_ (%)	LW at breath test (kg)	Mean LWG at breath test (g/d)
Control	Male	MC	24.3 ^abc^	9.3	45.1 ^a^	8.2 ^bcd^	17.5 ^bc^	25.0	15.8	581
Control	Male	KICA	54.4 ^c^	10.5	86.2 ^ab^	4.8 ^abcd^	14.2 ^abc^	30.5	15.0	391
Control	Female	MC	15.4 ^ab^	10.5	84.0 ^ab^	9.3 ^cd^	18.4 ^c^	30.6	14.1	485
Control	Female	KICA	38.9 ^bc^	7.6	58.1 ^a^	4.1 ^abc^	11.0 ^abc^	18.0	12.9	309
Ergot 5-high	Male	MC	26.9 ^abc^	7.1	57.9 ^a^	5.6 ^abcd^	11.9 ^abc^	18.6	12.3	344
Ergot 5-high	Male	KICA	30.3 ^abc^	7.2	124.6 ^b^	3.5 ^ab^	9.9 ^ab^	19.2	12.0	277
Ergot 5-high	Female	MC	23.6 ^abc^	11.2	50.3 ^ab^	8.7 ^cd^	14.8 ^abc^	21.6	10.2	180
Ergot 5-high	Female	KICA	42.5 ^c^	6.3	54.4 ^ab^	2.5 ^a^	8.2 ^a^	14.5	13.9	374
Ergot 15-high	Male	MC	11.3 ^a^	9.3	39.5 ^ab^	10.8 ^d^	19.0 ^c^	26.0	14.0	440
Ergot 15-high	Male	KICA	35.3 ^abc^	6.4	49.5 ^ab^	3.8 ^ab^	10.1 ^ab^	15.2	11.1	298
Ergot 15-high	Female	MC	21.9 ^abc^	10.3	63.2 ^ab^	8.6 ^cd^	16.6 ^abc^	27.7	13.3	345
Ergot 15-high	Female	KICA	34.8 ^abc^	11.9	48.9 ^ab^	6.5 ^abcd^	16.3 ^abc^	23.4	12.4	330
ANOVA (*p*-values)										
Diet	-	-	0.523	0.357	0.255	0.211	0.130	0.219	0.056	0.069
Sex	-	-	0.870	0.206	0.550	0.685	0.815	0.954	0.453	0.286
Substrate	-	-	0.002	0.206	0.271	0.001	0.024	0.171	0.594	0.176
Diet × sex	-	-	0.407	0.289	0.159	0.937	0.829	0.589	0.491	0.874
Diet × substrate	-	-	0.538	0.717	0.375	0.989	0.981	0.826	0.095	0.133
Sex × substrate	-	-	0.949	0.461	0.043	0.889	0.991	0.355	0.208	0.170
Diet × sex × substrate	-	-	0.522	0.091	0.709	0.235	0.277	0.352	0.549	0.592
PSEM	-	-	5.7	1.1	12.8	1.3	2.1	3.6	0.8	50

Abbreviations: LW = live weight, LWG = LW gain, *t*_max_ (min) = time at the maximum ^13^C-exhalation, DOB_max_ (‰) = maximum delta over baseline value, *t*_0.5 _(min) = time when the half of the recovered ^13^C is exhaled, cPDR_30, 60, 120_ (%) = cumulative percent of the applied ^13^C-dose recovery after 30, 60 or 120, MC = ^13^C-methacetin, KICA = ^13^C-ketoisocaproate; PSEM = pooled standard error of means.

**Table 6:** Correlation coefficients between various characteristics of weaned piglets fed a control diet or diets adjusted to a total ergot alkaloid content of 16.8 mg/kg diet (Ergot 5-high; Ergot 15-high) (week 3 of experiment, *n* = 24). Liver function was tested by the ^13^C-methacetin breath test.

	LW at breath test (kg)	Mean LWG, day of breath test (g/d)	Protein (g/L)	Albumin (g/L)	Total bilirubin (μmol/L)	Glucose (mmol/L)	ASAT (U/L)	ALAT (U/L)	GLDH (U/L)	GGT (U/L)	*t*_max _(min)	DOB_max_ (‰)	*t*_0.5_ (min)	cPDR_30_ (%)	cPDR_60_ (%)	cPDR_120_ (%)
LW at breath test (kg)	1.00	**0.85**	−0.10	0.13	0.40	−0.19	0.31	0.24	0.23	−0.04	0.38	−0.25	0.15	−0.16	0.08	0.15
Mean LWG, day of breath test(g/d)	-	1.00	−0.31	0.11	**0.51**	−0.12	**0.45**	0.20	**0.43**	0.00	0.07	−0.12	0.07	0.03	0.17	0.14
Protein (g/L)	-	-	1.00	0.24	−0.18	0.19	−0.17	−0.13	−0.29	0.10	0.19	−0.23	−0.08	−0.23	−0.19	−0.19
Albumin (g/L)	-	-	-	1.00	0.08	0.10	−0.04	0.25	0.25	**0.73**	0.11	−0.17	0.12	−0.11	0.00	0.07
Total bilirubin (μmol/L)	-	-	-	-	1.00	**−0.64**	**0.45**	0.07	0.28	0.06	−0.19	−0.20	0.34	−0.05	−0.01	−0.01
Glucose (mmol/L)	-	-	-	-	-	1.00	−0.22	0.17	0.10	0.12	0.10	−0.08	0.03	−0.05	−0.10	−0.06
ASAT (U/L)	-	-	-	-	-	-	1.00	0.40	0.29	−0.12	0.20	−0.25	0.09	−0.06	0.04	0.06
ALAT (U/L)	-	-	-	-	-	-	-	1.00	0.26	0.23	0.25	−0.23	0.28	0.00	0.06	0.18
GLDH (U/L)	-	-	-	-	-	-	-	-	1.00	0.35	−0.08	−0.12	0.04	0.13	0.16	0.13
GGT (U/L)	-	-	-	-	-	-	-	-	-	1.00	0.01	−0.19	0.18	−0.11	−0.09	0.02
*t*_max _(min)	-	-	-	-	-	-	-	-	-	-	1.00	−0.30	0.21	**−0.53**	−0.20	0.01
DOB_max _(‰)	-	-	-	-	-	-	-	-	-	-	-	1.00	−0.11	**0.73**	**0.67**	**0.57**
*t*_0.5 _(min)	-	-	-	-	-	-	-	-	-	-	-	-	1.00	−0.05	0.13	0.38
cPDR_30_ (%)	-	-	-	-	-	-	-	-	-	-	-	-	-	1.00	**0.91**	**0.77**
cPDR_60_ (%)	-	-	-	-	-	-	-	-	-	-	-	-	-	-	1.00	**0.94**
cPDR_120_ (%)	-	-	-	-	-	-	-	-	-	-	-	-	-	-	-	1.00

Abbreviations: LW = live weight, LWG = LW gain, GGT = Gamma-glutamyl transferase, ASAT = aspartat-aminotransferase, ALAT = alanine-aminotransferase, GLDH = glutamat-dehydrogenase, *t*_max_ (min) = time at the maximum ^13^C-exhalation, DOB_max_ (‰) = maximum delta over baseline value, t_0.5 _(min) = time when the half of the applied ^13^C is exhaled, cPDR_30, 60, 120 _(%) = cumulative percent of the applied ^13^C-dose recovery after 30, 60 or 120 min. Bold printed correlation coefficients are significantly different from zero (*p* < 0.05).

**Table 7:** Correlation coefficients between various characteristics of weaned piglets fed a control diet or diets adjusted to a total ergot alkaloid content of 16.8 mg/kg diet (Ergot 5-high; Ergot 15-high) (week 3 of experiment, *n* = 24). Liver function was tested by the ^13^C-ketoisocaproate breath test.

	LW at breath test (kg)	Mean LWG, day of breath test(g/d)	Protein (g/L)	Albumin (g/L)	Total bilirubin (μmol/L)	Glucose (mmol/L)	ASAT (U/L)	ALAT (U/L)	GLDH (U/L)	GGT (U/L)	*t*_max_ (min)	DOB_max_ (‰)	*t*_0.5 _(min)	cPDR_30_ (%)	cPDR_60_ (%)	cPDR_120_ (%)
LW at breath test (kg)	1.00	**0.73**	−0.14	0.44	0.05	0.13	0.03	0.16	0.23	−0.02	0.04	0.23	−0.04	0.29	0.33	0.30
Mean LWG, day of breath test (g/d)	-	1.00	**−0.51**	**0.54**	0.35	−0.03	0.15	0.32	0.46	0.37	0.28	0.13	−0.40	0.01	0.19	0.11
Protein (g/L)	-	-	1.00	−0.22	−0.25	0.28	−0.05	−0.39	−0.21	−0.52	0.24	0.12	0.01	−0.01	0.03	0.20
Albumin (g/L)	-	-	-	1.00	0.26	0.07	**−0.48**	−0.21	0.45	0.17	−0.02	0.41	−0.19	0.41	**0.49**	0.38
Total bilirubin (μmol/L)	-	-	-	-	1.00	**−0.50**	−0.03	−0.29	0.35	−0.03	0.12	0.21	−0.15	0.02	0.14	−0.01
Glucose (mmol/L)	-	-	-	-	-	1.00	−0.37	−0.08	−0.27	−0.01	0.21	0.03	0.22	−0.08	0.02	0.38
ASAT (U/L)	-	-	-	-	-	-	1.00	**0.61**	−0.13	0.01	0.08	−0.40	−0.26	−0.28	−0.35	−0.45
ALAT (U/L)	-	-	-	-	-	-	-	1.00	0.04	0.22	0.09	−0.43	−0.10	−0.35	−0.38	−0.29
GLDH (U/L)	-	-	-	-	-	-	-	-	1.00	0.43	−0.04	−0.14	−0.35	0.12	0.05	−0.13
GGT (U/L)	-	-	-	-	-	-	-	-	-	1.00	−0.16	−0.35	−0.08	−0.17	−0.27	−0.33
*t*_max_ (min)	-	-	-	-	-	-	-	-	-	-	1.00	0.21	−0.53	**−0.51**	−0.06	0.25
DOB_max_ (‰)	-	-	-	-	-	-	-	-	-	-	-	1.00	−0.26	**0.57**	**0.84**	**0.64**
*t*_0.5 _(min)	-	-	-	-	-	-	-	-	-	-	-	-	1.00	0.03	−0.12	0.11
cPDR_30_ (%)	-	-	-	-	-	-	-	-	-	-	-	-	-	1.00	**0.85**	**0.50**
cPDR_60_ (%)	-	-	-	-	-	-	-	-	-	-	-	-	-	-	1.00	**0.78**
cPDR_120_ (%)	-	-	-	-	-	-	-	-	-	-	-	-	-	-	-	1.00

Abbreviations: LW = live weight, LWG = LW gain, GGT = γ-glutamyl transferase, ASAT = aspartat-aminotransferase, ALAT = alanine-aminotransferase, GLDH = glutamat-dehydrogenase, *t*_max_ (min) = time at the maximum ^13^C-exhalation, DOB_max_ (‰) = maximum delta over baseline value, *t*_0.5 _(min) = time when the half of the applied ^13^C is exhaled, cPDR_30, 60, 120, 180_ (%) = cumulative percent recovery of the applied ^13^C-dose after 30, 60 or 120 min. Bold printed correlation coefficients are significantly different from zero (*p* < 0.05).

### 2.5. Correlations

Individual data of the MC and KICA breath test and of clinical-chemical characteristics were used to construct correlation matrices (Table 6 and Table 7). Various breath test parameters and serum clinical-chemicals were significantly correlated to each other, while the correlations between breath test data and serum clinical chemical characteristics were rather weak.

## 3. Discussion

It has been repeatedly pointed out that an efficient protection of farm animals from ergot, which might be present in feed, can only be achieved when the toxic compounds, *i.e.*, the ergot alkaloids, are analyzed in feed and related to the health and performance of animals. Only by collecting such corresponding data will it be possible to replace the current regulation on the upper limit of 1000 mg ergot per kg unground cereal grains. Thus we aimed at this issue with a special focus on the possible effects of ergot alkaloids on the porcine liver.

Although the TA content of the ergot-containing diets varied from 9 to 22 mg/kg the effects on performance appeared to be independent of both the ergot source and the TA content. However, it needs to be stressed that especially the piglets fed the Ergot 5-high diet, which contained 22 mg TA/kg, were characterized by the lowest feed intake compared to piglets fed all the other ergot-containing diets (9–14 mg TA/kg). Live weight gain over the whole experiment of the latter three groups was 28% to 35% lower than that of the control group, while group Ergot 5-high gained 46% less live weight than the control group, which is equivalent to an additional drop of 11% to 18% compared to the other three ergot fed groups. Due to the variation and the limited number of replications, the differences failed to reach significance. However, putting the data of the present experiment together within the frame of literature findings, a clear linear negative relationship between the dietary TA content and performance of rearing piglets becomes obvious [20]. Based on this literature compilation, it can be deduced that feed intake, live weight gain and gain-to-feed ratio decrease by 0.9%, 1.24% and 0.9%, respectively, per incremental increase of 1 mg TA/kg diet. 

Besides the effects of ergot alkaloids on the gross performance of the piglets, a second aim of the present experiment was to evaluate possible liver effects. Earlier findings revealed that ergot alkaloids affected the nutrient status of the liver [2,3,4]. Moreover, hepatic CYP metabolism of and induction by ergot alkaloids have been demonstrated [5,6]. Additionally, the hepatic mitochondrial Ca^2+^ ATPase activity was shown to be influenced by ergot alkaloids [7]. Thus, we hypothesized that ergot alkaloids fed to piglets affect the liver at different subcellular compartments. In order to test this hypothesis, we used KICA and MC as breath test substrates to evaluate the effects of ergot alkaloids on hepatic branched-chain α-ketoacid dehydrogenase (BCKDH, EC 1.2.4.4) and on CYP1A2 (EC 1.14.14.1), respectively.

A literature review about advantages and pitfalls of breath tests for evaluation of the liver functional capacity in general, and of hepatic mitochondrial function in particular, suggested that KICA and methionine are promising substrates [12]. While the complex metabolic pathways of methionine still have to be studied, with regard to the most appropriate label position, the metabolism of KICA undergoes two main enzymatic pathways, which include the transamination into the corresponding branched-chain amino acid leucine and the oxidative decarboxylation by BCKDH [12]. The latter enzyme is located in mitochondria, and the main activity is confined to the liver of humans (for reviews see [11,12]; rats [21] and pigs [22]). The dysfunction of hepatocyte mitochondria is regarded as one of the earliest signs of a dysfunction of the whole organ, as damage to this cellular structure occurs at an earlier stage than in the whole hepatocyte [23]. Thus, testing this decarboxylase using ^13^C breath tests is thought to be a tool for detecting effects of xenobiotics [12,23]. Among other factors, the liver specificity of the KICA breath test depends on the quantitative proportion of the hepatic BCKDH activity of the total body activity. For pigs, there is experimental evidence about this proportion. It could be demonstrated that liver was characterized by an approximately forty-times higher total activity than muscle when expressed per gram tissue, and it was still eighteen times higher when related to hepatic protein. However, when the total masses of liver and muscles were considered, the contribution of the liver was just 2.3-times higher than that of the muscles [22]. Interestingly, excessive dietary leucine supply (approximately 50% higher than the requirement) resulted in a significant stimulation of the BCKDH activity both in the liver and in the muscle. For a human KICA breath test, it is generally recommended to supply 20 mg/kg of leucine per person orally in order to prevent or to minimize the transamination pathway of KICA and to force the label to the terminal and irreversible degradation pathway through the BCKDH activity with the intended CO_2_ formation. Whether the leucine stimulatory effect on the BCKDH activity interferes with the hepatic effects to be evaluated with the KICA breath test has not been addressed thus far. Originally, the KICA breath test was performed without the supplemental leucine [21,24] and could be demonstrated to be sensitive for detection of xenobiotic effects. Later, the KICA was given to humans in the absence and presence of increasing amounts of leucine, and it could be shown that the label recovery (cPDR) was higher in the presence of leucine. However, in spite of the different label recovery, there was a clear correlation of both tests within subjects [25]. Thus, the detection of treatment effects on BCKDH activity is also possible without supplemental leucine. 

In contrast to KICA, the substrate MC is metabolized by the microsomal mixed functional oxidase P4501A2, which exhibits its main activity in the liver, and the clinical relevance of this test lies in the assessment of the hepatic residual functional microsomal mass (for review see [11]). With regard to the relevance of this P450 isoform, it was deduced from literature findings that all main activities of human CYP isoforms were also found in porcine liver microsomes [26,27]. Although no information is available on the total proportion of porcine hepatic P4501A2 of total body activity, the total P450 activity in liver microsomes is considered to be comparable for humans and pigs [28]. Based on these assumptions, the applicability of the MC breath test to evaluate toxin effects on the porcine liver has been proposed [29].

The liver specificity of both breath tests also depends on the hepatic extraction rate (difference between hepatic substance inflow and outflow concentration divided by the inflow concentration) of the test substrates in relation to blood flow [11]. Ideal substrates should be characterized by low hepatic extraction rates, which make the test less dependent on hepatic blood flow. Both test substrates used in the present experiment are efficiently extracted by the liver. MC is regarded as a high extraction rate substance (>0.8) in humans [11], while the KICA hepatic extraction rate amounts to approximately 0.6 in rats [30]. Thus, differences in blood flow, caused either by individual variation or by treatment effects, need to be considered with regard to overall variation in discussing the results. General effects of ergot alkaloids on blood circulation could be expected based on their potential interactions with adrenergic, serotoninergic and dopaminergic receptors [1]. However, in the view that breath test results did not significantly differ in ergot-exposed piglets from the control piglets, there were obviously no adverse effects both on the tested mitochondrial and microsomal functions and on hepatic blood flow.

The significant interactions between breath test substrate and sex were mainly caused by the significantly prolonged elimination half-life (*t*_0.5_) in male piglets fed the Ergot 5-high diet when tested with KICA compared to all other sub-groups independent of test substrate, treatment group or sex. The reasons for this isolated prolongation cannot be explained by other parameters recorded in the experiment. As a consequence of the prolonged tracer elimination, these piglets exhibited the lowest cPDR. Other piglets also tested with KICA similarly showed a lower cPDR, particularly during the first hour after the administration of the substrate. This initially retarded tracer recovery is also reflected by the approximately 18 min delayed tracer climax (*t*_max_) estimated for the KICA breath test. These marked differences were probably not caused by differences in gastric emptying, as both test substrates were administered via a probe directly into the stomach. The smoother increase in KICA oxidation (see Figure 1) and the later *t*_max_ might be caused by at least two reasons. Firstly, the mitochondrial and microsomal kinetics of KICA and MC oxidation, respectively, might be different due to the underlying differences in metabolic pathways and biochemical events at these subcellular fractions. Secondly, a part of the labeled KICA might temporarily be retained as labeled leucine via the transamination pathway and a subsequent leucine oxidation, which again occurs via KICA. Evidence for the latter explanation comes from human and rat breath test studies employing both ^13^C-KICA and ^13^C-leucine as test substrates [25]. Leucine oxidation climaxed approximately 15 to 20 min later than KICA (55 *vs. *35 min in males and 50 *vs.* 35 min in females). A similar delay in *t*_max_ for oxidized leucine relative to KICA has been observed in rats [31]. Moreover, hypothyroid rats used in these experiments showed decreased energy expenditure and an increased KICA and leucine oxidation as measured by ^13^C-breath tests with substrates administered intravenously. These results further indicate that the thyroid status obviously influences the leucine pools, the protein turnover and, finally, the degree of KICA transamination and decarboxylation rate and might consequently contribute to the overall variation as observed with the KICA breath test. 

Although feeding the diet Ergot 5-high with the higher TA content of 22 mg/kg tended to decrease the cPDR_60_ by 26% and 28% in the MC and KICA breath test, respectively, this difference to the control groups failed to reach significance. Furthermore, feeding the Ergot 15-high diet with a TA concentration of 13.5 mg/kg apparently did not differ from the corresponding control groups, both according to the MC and KICA breath test, respectively (−1% *vs.* +5%). Therefore, both tests discriminated between treatments to the same extent, and the missing significance of the interactions between dietary treatment and test substrate underlines this conclusion.

In reviewing the usefulness of ^13^C breath tests for diagnosing liver fibrosis, it was concluded that a significant proportion of patients suffering from chronic viral hepatitis and a few suffering from non-alcoholic fatty liver disease were characterized by normal aminotransferase levels despite significant hepatic lesions [32]. Therefore, it was also of interest for the present experiment to correlate the serum-clinical parameters more or less indicative for liver health with the results of the breath tests (Table 6 and Table 7). With the exception of serum albumin concentration in the KICA breath test, none of the other parameters correlated with the results of the breath test. Therefore, the variation observed in the breath test results cannot be explained by variation in the GGT, ALAT, ASAT and GLDH activities in serum. As no correlation was observed between the serum albumin concentration and the breath test results according to the MC breath test, the significant medium positive correlation in the KICA breath test is difficult to explain. 

Significant treatment effects were observed for the serum bilirubin concentration. Among others, the total bilirubin level in serum is determined by the balance between the degree of hemoglobin degradation and its elimination with the bile via the liver. Therefore, increased total bilirubin concentrations might result from an increased hemolysis and/or a compromised hepatic bile acid formation, conjugation and elimination. However, neither a cholestatic condition nor other hepatocellular damages as indicated by the unaltered GGT and the GLDH and ALAT activity, respectively, were detected. In addition, the results of the breath tests further substantiate the view that the increased total bilirubin concentrations were probably not related to the liver bile formation process, but might be associated to the overnight starvation of the piglets before blood was collected and the breath test was performed. This view is supported by the negative correlation of −0.5 between total bilirubin and glucose concentration, which partially caused the significant group differences for the serum glucose concentrations (Table 6 and Table 7). As a group feeding system was used for piglet keeping, the individual starvation times could only partially be controlled. The higher feed intake level of the control group might explain why these piglets responded more sensitively than ergot-fed piglets, which were generally characterized by a lower feed intake (Table 3).

## 4. Experimental Section

### 4.1. Experimental Design

Two ergot batches were used in the experiment. While Ergot Batch 5 (Ergot 5) contained 100% ergot, the Ergot Batch 15 (Ergot 15) was composed of 45.2% ergot and 54.8% rye. The latter batch contained approximately 30% less total alkaloids than Ergot 5. Moreover, the batches differed most markedly in the ergotamine, ergotaminine, ergocristine, ergocryptinine and ergosine content (Table 1). Considering the differences in total alkaloid contents, both batches were mixed into piglet diets in such a way as to provide constant total target alkaloid contents of 8.4 mg/kg (Ergot 5-low, Ergot 15-low) and 16.8 mg/kg (Ergot 5-high, Ergot 15-high) (Table 2). The adjustment of two ergot batches, which were characterized both by different total alkaloid contents and varying alkaloid patterns, to similar dietary total alkaloid contents aimed at investigating the effects of the variance caused by the differences in the alkaloid composition. Thus, diets were prepared without ergot (Control) or containing 8.4 mg total alkaloids/kg diet (Ergot 5-low, Ergot 15-low) and 16.8 mg total alkaloids/kg diet (Ergot 5-high, Ergot 15-high) (Table 2).

### 4.2. Experimental Procedure

A total of 40 castrated male and 40 female piglets, weaned at an average age of 21 d, were assigned to 20 slatted floor pens (2 male and 2 female piglets per pen), located in an air-conditioned experimental stable. Each of the 5 experimental diets (Table 1) was tested on 16 piglets distributed in 4 floor pens. The experiment started after a period of 10 d in which piglets were fed a commercial diet. The mean body weights for piglets fed diets Control, Ergot 5-low, Ergot 5-high, Ergot 15-low and the Ergot 15-high were 8.1 ± 1.1 kg, 8.2 ± 1.3 kg, 8.2 ± 1.2 kg, 8.1 ± 1.0 kg and 8.2 ± 1.0 kg, respectively, at the beginning of the experiment. The experimental period covered 5 weeks and body weight and feed consumption were recorded on a weekly basis. Diets were provided as a meal for *ad libitum* consumption, while water was offered via nipple drinkers.

For evaluation of the liver function, a breath test using both MC and KICA was performed in the fourth week of the experiment after a 12 h period of feed deprivation. For this purpose, KICA and MC (EURISO-TOP GmbH, Saarbrücken, Germany) were dissolved in tap water. The solutions were directly placed into the stomach of the piglets at a dose of 2 mg MC or KICA/kg body weight with the aid of a duodenal feeding tube, according to Levin (125 cm CH 18 B.Braun Melsungen AG D-34209 Melsungen, Germany). In total, 48 piglets were used for the breath test. Only the groups Control, Ergot 5-high and the Ergot 15-high were tested. Within each of these breath test groups, 8 female and 8 male piglets were used, and half of each sex was given MC or KICA as test substrate (*n* = 4).

Before the test substrates were administered, a zero breath sample was collected. For collecting breath, a mask designed for small animals (Jørgen Kruuse A/S DK-5290 Marslev, Denmark) was used. The mask was connected via a two-way valve to an aluminum coated breath sampling bag (Wagner Analysen Technik Vertriebs-GmbH, Bremen, Germany). Further breath samples were collected at 15, 30, 45, 60, 75, 90, 105 and 120 min after substrate administration. Our earlier experiments have shown that a total collection time of 120 min is sufficient to discriminate between treatments and that a prolongation of the sampling time does not increase the statistical power. Therefore, the total test time was restricted to 120 min. 

Blood samples were drawn by puncture of the large neck vessels after collecting the zero breath sample for determination of clinical-chemical characteristics from all piglets used in the breath test (8 female and 8 male piglets per treatment). 

Treatments and experiment were conducted according to the European Community regulations concerning the protection of experimental animals and were approved by the Land Bureau for Consumer Protection and Food Safety for Lower Saxony (LAVES) in Oldenburg, Germany (File Number 509.42502/09-02.02).

### 4.3. Analyses

#### 4.3.1. Ergot and Feed

Ergot batches and diets were examined for dry matter, crude protein, crude ash, crude fat, starch and sugar according to the official standard methods of the Association of German Agricultural Research and Investigation Institutions (VDLUFA) [33]. Analysis of ergot alkaloids (ergometrine, ergocornine, ergotamine, α-ergocryptine, ergosine, ergocristine and their -inine isomers) in ergot batches and diets was performed with an HPLC based method [34], as described in detail elsewhere [35]. The detection limit amounted to 5 ng/g, except for ergometrine, where it was 10 ng/g at a sample size of 5 g. The mean recovery rate of the alkaloids was 79%. The results of the analyses were not corrected for recovery. Ergometrine, ergotamine, ergocristine, ergocornine and ergocryptine are referred to as “key alkaloids”, as standards were commercially available for their identification. Ergosine and its isomer were identified by their retention time [36]. The sum of all identified alkaloids (-ine and -inine isomers) is termed as total alkaloids.

Ergot batches were additionally analyzed for fatty acids by gas chromatography, as described in detail earlier [37,38].

#### 4.3.2. Breath Samples

The infrared ^13^C isotope spectrometer IRIS (IRIS, Wagner Analysen Technik GmbH, D-28357, Bremen, Germany) was used for determining the ^13^C/^12^C ratios. The measured ^13^C/^12^C ratios were expressed as the relative difference from the Pee Dee Belemnite limestone carbon reference standard. Moreover, the so calculated delta values were expressed as the difference from the basal delta value before the tracer administration, *i.e.*, as delta over base line (DOB).

#### 4.3.3. Clinical-Chemical Characteristics

Activities of glutamate dehydrogenase (GLDH), Gamma-glutamyltransferase (GGT), alanine-aminotransferase (ALAT) (Labor + Technik, Eberhard Lehmann, Berlin, Germany) and aspartate aminotransferase (ASAT) (Labor + Technik, Eberhard Lehmann, Berlin, Germany, opt. DGKC) were measured in serum by enzymatic UV-standard procedures. Protein and albumin concentrations were determined using the biurette method and a colorimetric test with bromcresol green, respectively. Analyses were performed at the Clinic for Swine and Small Ruminants, Veterinary School, Hannover, Germany.

### 4.4. Calculations and Statistics

#### 4.4.1. Breath Test

The DOB values were fitted to a non-linear regression for modeling the kinetics of the metabolized MC [39]:

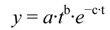
(1)
where *y* = DOB (‰), *t* = time after the bolus (min), and *a*, *b* and *c* are regression coefficients.

The time at the maximum ^13^C-exhalation (*t*_max_), the maximum DOB (DOB_max_) and the time when the half of the recovered ^13^C was exhaled (*t*_0.5_) could be estimated from the above regression coefficients.

The ^13^C-recovery with breath is expressed as percentage of the applied ^13^C-dose (PDR = percentage dose recovery). The cumulative ^13^C-recovery (cPDR = cumulative percentage dose recovery) is calculated from the PDR and indicates the sum of the detected ^13^C. Details of calculations are described elsewhere [29]. 

#### 4.4.2. Analysis of Variance (ANOVA) and Further Statistics

Live weight gain and serum clinical-chemical characteristics were analyzed according to a complete two-by-two factorial design of ANOVA with dietary treatment, sex and their interactions as fixed effects. Although 16 piglets were tested per treatment, the recorded live weight gain and other performance data were expressed on a mean box-basis (*i.e.*, *n* = 4), as feed intake and feed-to-live weight gain data could only be evaluated on a box-basis for technical reasons (one automatic feeder for 4 free-ranging piglets). Consequently, feed intake and feed-to-gain ratio were evaluated according to a one-way ANOVA, with dietary treatment being the only fixed effect. Due to inclusion of two substrates used in the breath test, data could be analyzed according to a complete three factorial ANOVA with dietary treatment, sex and substrate, as well as their interactions, as fixed effects. 

Significant differences between means were evaluated by the Student-Newman-Keuls-test (*p *< 0.05).

Pearson correlation coefficients were calculated and used to construct a correlation matrix for all considered parameters (clinical-chemical characteristics, breath test results).

All statistics were carried out using the Statistica for the WindowsTM operating system [40].

## 5. Conclusions

Ergot alkaloids decrease the feed intake and live weight gain of piglets markedly, while the effects on the liver as evaluated by breath tests and by serum clinical chemical characteristics are less pronounced.

Methodologically, future research should consider the detailed investigation of the effects of various doses of supplemental leucine on the detectability of dietary treatment effects to be evaluated by the KICA breath test.
